# A new genus and two new species of miniature clingfishes from temperate southern Australia (Teleostei, Gobiesocidae)

**DOI:** 10.3897/zookeys.864.34521

**Published:** 2019-07-15

**Authors:** Kevin W. Conway, Glenn I. Moore, Adam P. Summers

**Affiliations:** 1 Department of Wildlife and Fisheries Sciences and Biodiversity Research and Teaching Collections, Texas A&M University, College Station, TX 77843, USA; 2 Research Associate, Ichthyology, Australian Museum Research Institute, 1 William Street, Sydney, NSW 2010, Australia; 3 Fish Section, Department of Aquatic Zoology, Western Australian Museum, Locked Bag 49 Welshpool DC WA 6986, Australia; 4 School of Biological Sciences, University of Western Australia, Nedlands, WA 6907, Australia; 5 Friday Harbor Laboratories, University of Washington, Friday Harbor, Washington 98250, USA; 6 Burke Museum of Natural History and Culture, University of Washington, Seattle, WA 98105, USA

**Keywords:** Macroalgae, miniaturisation, osteology, reduction, taxonomy

## Abstract

A new genus and two new species of miniature clingfishes are described based on specimens collected from dense stands of macroalgae in intertidal and shallow subtidal areas along the coast of southern Australia. The new genus, *Barryichthys*, is distinguished from other genera of the Gobiesocidae by unique features of the adhesive disc, including elongate papillae in adhesive disc regions A and B, the reduction and/or loss of several elements of the cephalic lateral line canals, the lower gill arch skeleton, and the neurocranium, and by having two distinct types of pectoral-fin rays. *Barryichthyshutchinsi* is described based on 19 specimens (12.4–18.7 mm SL) from Western Australia and South Australia. *Barryichthysalgicola* is described based on 22 specimens (9.0–21.0 mm SL) from Victoria, New South Wales and Tasmania. The new species are distinguished from each other by characters of body and head shape, vertebral counts, and aspects of live colour pattern. The new genus shares several characters in common with *Parvicrepis*, another genus of miniature gobiesocids from southern Australia that also inhabits macroalgae habitats. The many reductions and novel characters of *Barryichthys* are discussed within the context of miniaturisation.

## Introduction

The family Gobiesocidae contains 50 genera and more than 170 species of predominately marine fishes found in coastal areas of the Atlantic and Indo-Pacific oceans, from the intertidal zone to ~500 meters depth (Briggs 1955; Hastings and Conway 2017). Seven species are known to inhabit freshwater streams in the Neotropics (Briggs and Miller 1960; Conway et al. 2017a). Commonly referred to as clingfishes, members of this family generally exhibit a well-developed ventral adhesive disc (formed by elements of the paired fins and paired-fin girdles; Guitel 1888), with which they can attach to smooth or even heavily structured substrates with great tenacity (Wainwright et al. 2013; Ditsche et al. 2014). Although some clingfishes may reach body lengths over 200 mm in standard length (SL) (e.g., *Sicyasessanguineus* Müller & Troschel in Müller 1843), the majority are small-bodied and do not exceed 50 mm SL (Briggs 1955; Brandl et al. 2018). Several small-bodied clingfishes are not known to exceed 26 mm SL and are considered miniature species following the criteria of Weitzman and Vari (1988).

A number of temperate species of clingfishes, including several small-bodied or miniature species, are known to exhibit intimate (potentially obligate) associations with macroalgae and/or seagrasses. This includes members of the genus *Rimicola* Jordan and Evermann in Jordan, 1896 in the western Pacific (Roland 1978; Lamb and Edgell 2010), *Acyrtops* Schultz, 1951 in the western central Atlantic (Gould 1965), *Opeatogenys* Briggs, 1955 in the eastern central Atlantic (Hofrichter and Patzner 2000; Gonçalves et al. 2005), *Eckloniaichthys* Smith, 1942 in South Africa (Allen and Griffiths 1981), *Parvicrepis* Whitley, 1931, *Posidonichthys* Briggs, 1993, and two species of *Cochleoceps* (*C.spatula* (Günther, 1861) and *C.viridis* Hutchins, 1991) in southern Australia (Briggs 1993; Hutchins 1983, 1991, 1994a, 2008), and *Gastrocyathus* Briggs, 1955, *Gastrocymba* Briggs, 1955, *Gastroscyphus* Briggs, 1955, and *Haplocylix* Briggs, 1955 in New Zealand (Paulin and Roberts 1992; Stewart 2015). All these taxa share a number of characteristics that may represent adaptations for dwelling on the surface of macroalgae and/or seagrass blades, including narrow, elongate bodies and relatively narrow heads, short dorsal and anal fins, modified pectoral fins in which the lower rays are generally notably shorter than the upper rays (Briggs 1955), and live colour patterns comprised predominately of different shades of green, brown, orange or red. This type of colouration likely facilitates crypsis on the fronds of macroalgae or blades of seagrass to which they adhere (Paulin and Roberts 1992; Hofrichter and Patzner 2000).

Several undescribed species of macroalgae and/or seagrass inhabiting clingfishes have been known from the southern coast of Australia since at least the 1980s (Hutchins 1983, 1991a, b; Last et al. 1983; Kuiter 1993). They are considered to represent at least four different genera, three of which have yet to be formally described (viz. Genus A, B, and C sensu Hutchins 1994a, 2008). Hutchins (1994a, 2008) considered the undescribed Genus B to be monotypic and comprised of a single undescribed species (referred to using the common name “Rat Clingfish”; Hutchins 1991b, 1994a, 2008) with a disjunct distribution in shallow coastal areas along the southern coast of Australia, including Western Australia in the west and Victoria and Tasmania in the east (Hutchins 2008). Members of Genus B are very small (≤21 mm SL) and similar in general appearance to members of *Parvicrepis*, with which they are sympatric in shallow coastal areas rich in “weed” (Hutchins 1994a, 2008). Examination of unidentified and unsorted material of gobiesocids as well as material identified previously as *Parvicrepis*, from the southern coast of Australia held within the Western Australian Museum (Perth) and the Australian Museum (Sydney) produced additional specimens of the undescribed Genus B for study. Based on differences in vertebral counts, body and head shape, and colouration in life, we consider this material of Genus B to represent two different species, both of which are undescribed. In the present paper, we provide descriptions for these two new miniature species, and provide a formal description for the undescribed Genus B, which we name *Barryichthys* gen. nov.

## Materials and methods

Specimens used in this study were obtained from the following museum collections: Australian Museum, Sydney (**AMS**); Biodiversity Research and Teaching Collections, Texas A&M University, College Station (**TCWC**); and Western Australian Museum, Perth (**WAM**). Head and body measurements and counts reported follow Conway et al. (2014) and are expressed as percent of standard length (SL) or head length (HL). Adhesive disc papillae terminology follows Briggs (1955) and Hutchins (2008). Cephalic lateral line pore terminology follows Shiogaki and Dotsu (1983), except that we also use numbers to refer to individual pores following Conway et al. (2017b), with pores numbered along a particular canal from anterior to posterior or dorsal to ventral. General osteological terminology follows that of Springer and Fraser (1976), except that we use the term anguloarticular instead of articular, anterior ceratohyal instead of ceratohyal, autopalatine instead of palatine, epicentral instead of epipleural (following Gemballa and Britz 1998), endopterygoid instead of mesopterygoid, pharyngobranchial instead of infrapharyngobranchial, posterior ceratohyal instead of epihyal, and retroarticular instead of angular.

Selected specimens were cleared and double stained (C&S) for bone and cartilage investigation using the protocol of Taylor and Van Dyke (1985). Computed tomography (CT) scans of select specimens were also obtained at the Karel F. Liem BioImaging Center (Friday Harbor Laboratories, University of Washington) using a Bruker (Billerica, MA) SkyScan 1173 scanner with a 1 mm aluminium filter at 60 kV and 110 μA on a 2240 x 2240 pixel CCD at a resolution of 8.8 μm. Specimens were scanned simultaneously in a 50ml plastic Falcon tube (Corning, NY), in which they were wrapped with cheesecloth moistened with ethanol (70%) to prevent movement during scanning. The resulting CT data were visualised, segmented, and rendered in Horos (www.horosproject.org) and Amira (FEI). Select specimens were reversibly stained using cyanine blue following Saruwatari et al. (1997) to aid examination of adhesive disc papillae and cephalic lateral line canal pores. Specimens or parts thereof were observed and photographed using a ZEISS SteREO Discovery V20 stereomicroscope equipped with a ZEISS Axiocam MRc5 digital camera. Digital images were typically stacked using ZEISS Axiovision software. All digital images were processed using Adobe Photoshop and Adobe Illustrator.

## Taxonomy

### Barryichthys gen. nov.

Taxon classificationAnimaliaGobiesociformesGobiesocidae

http://zoobank.org/505099BF-E797-43FC-BEC0-B433A0398707

Genus B Hutchins 1994a: 309; 2008: 725.

#### Diagnosis.

**Figure 1.**
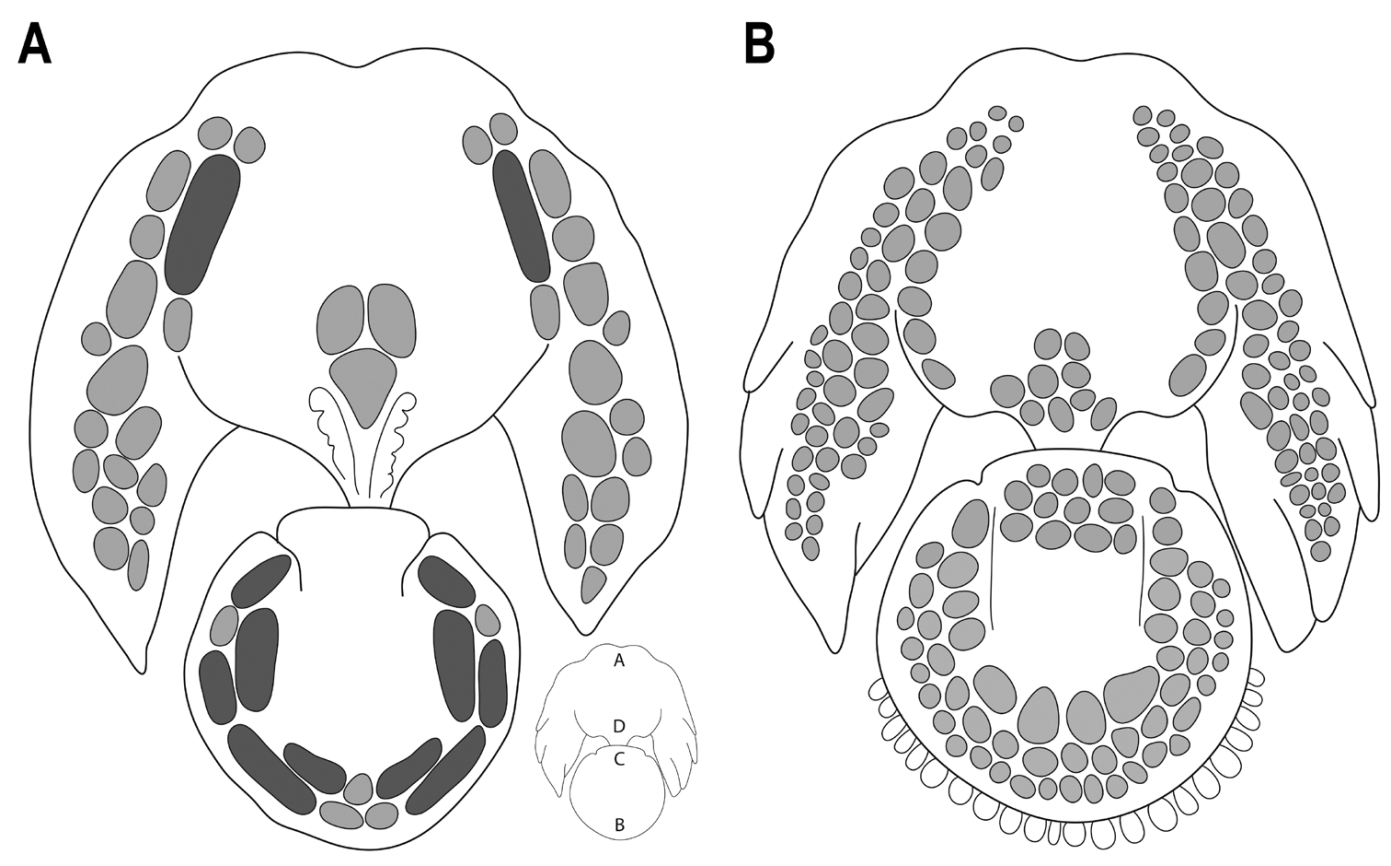
Schematic outline drawings of the adhesive disc of *Barryichthys* (**A**) and *Parvicrepis* (**B**). Both redrawn from Hutchins (1994: fig. 1). Typical circular-cuboid papillae in light grey (**A** and **B**); elongate papillae in dark grey (**A**). Disc regions A–D shown in inset figure.

A genus of the Gobiesocidae differing from all other genera by the following unique characters: a double adhesive disc with elongate papillae in regions A and B (Fig. 1A), few enlarged papillae (with circular or elongate cuboid margins) in disc region D, and papillae absent from region C; two distinct types of ray in the pectoral fin including a longer ray comprising a pair of poorly ossified and unsegmented hemitrichia (uppermost 10–12 rays) and a shorter, stouter ray comprising a pair of well-ossified and segmented hemitrichia (lowermost 4–5 rays); anterior part of parasphenoid a narrow strut of bone, ~1/4 width of wider posterior part of bone; a greatly reduced gill-arch skeleton in which the hypobranchial and basibranchial elements (including cartilages) and lower pharyngeal jaw teeth are absent; and a sexually dimorphic urogenital papilla that is housed within a shallow groove posterior to the anus that is either flanked by a pair of swollen skin folds (male) or not (female). The following characters are also diagnostic, although not unique to the genus: a well-developed skin pad covering base of lower pectoral-fin rays and girdle; a thick, fleshy upper lip that is thicker along midline than at lateral margins; the absence of preoperculo-mandibular and lachrymal lateral line canals; a single lateral line canal pore (PO1) posterior to orbit; gill filaments of the first gill arch comprising a hemibranch of 5–6 poorly developed gill filaments; branchiostegal rays 5 or 6; dorsal and anal fins with 4–6 rays, well separated from caudal fin; 4+4 principal caudal-fin rays; and 1–2 procurrent caudal-fin rays.

#### Etymology.

Named for Barry Hutchins, in honour of his work on Australian clingfishes. Masculine.

#### Type species.

*Barryichthyshutchinsi* sp. nov.

#### Remarks.

Hutchins (1994a) provided a brief overview of *Barryichthys* (his Genus B) based on relatively few specimens from the coasts of Victoria and Tasmania. Later, Hutchins (2008) provided a more in-depth summary of the characteristics that he considered important for separating his Genus B from other genera of gobiesocids inhabiting the southern coast of Australia and extended the range of the genus to Western Australia.

### Barryichthys hutchinsi sp. nov.

Taxon classificationAnimaliaGobiesociformesGobiesocidae

http://zoobank.org/DE65B196-C878-4524-850E-1DA1C8CB3548

Figs 2A , 3A–C , 4A , 5A , 6 , 7 , 8 , 9A, C–E , 10

Common name: Brown rat clingfish
Genus B sp. Hutchins 2008: 725.

#### Holotype.

WAM P.28981-004, male, 15.4 mm SL; Western Australia, Cottesloe Reef platform, Perth (31°59'00.0"S, 115°45'00.0"E), 16 January 1986, J. Keesing et al., CT scan: https://doi.org/10.17602/M2/M78748.

#### Paratypes.

*Western Australia*: WAM P.28981-003, 4, 16.0–16.9 mm SL; same data as holotype. – WAM P.34510-001, 5 (2 C&S), 14.2–16.3 mm SL; Western Australia, Cottesloe Reef platform, Perth (31°58'59"S, 115°45'00"E), 29 January 1985, J. Keesing. – WAM P. 34940-001, 1, female, 18.7 mm SL; Trigg Reef platform, Perth (31°52'46.5"S, 115°45'04.7"E), 13 January 1986, J. Keesing et al.

#### Other material.

*South Australia*: AMS I.20171-012, 6 (2 C&S), 12.4–13.1 mm SL (immature); South Australia; Kangraoo Island, Vivonne Bay (36°00'00.0"S, 137°10'48.0"E), D. Hoese & K. Handley. – AMS I.49000-001, 2 (1 CThttps://doi.org/10.17602/M2/M80016), 14.0–14.6 mm SL; Victor Harbor, Bluff Jetty (35°35'19.1"S, 138°36'16.5"E), 25 March 2015, G. Short.

#### Diagnosis.

*Barryichthyshutchinsi* is distinguished from *B.algicola* (below) by a shorter, deeper body (body depth at dorsal-fin origin 10–11% SL vs. 7–8% SL), a wider, deeper head (head width at widest point 66–75% HL vs. 55–61%; depth at orbit 30–32% HL vs. 27–29%; interorbital width 27–33% HL vs. 20–24%), ventral margin of the orbit obscured by cheek in ventral view (vs. entire ventral margin of orbit visible in ventral view), by having a shorter abdominal region with fewer vertebrae (abdominal vertebrae 17 vs. 21) and fewer ribs (11–12 vs. 15), fewer epicentrals (14–15 vs. 18–19), and a lower total number of vertebrae (total number of vertebrae 38–39 vs. 42–44), and by features of live colour pattern, including body background colour golden-yellow to olive-brown (vs. uniform green), the presence (vs. absence) of a variable number of irregularly shaped light to dark brown markings along dorsal midline, and the presence (vs. absence) of a series of light to dark brown elongate lateral markings forming an incomplete or complete horizontal stripe.

#### Description.

**Figure 2.**
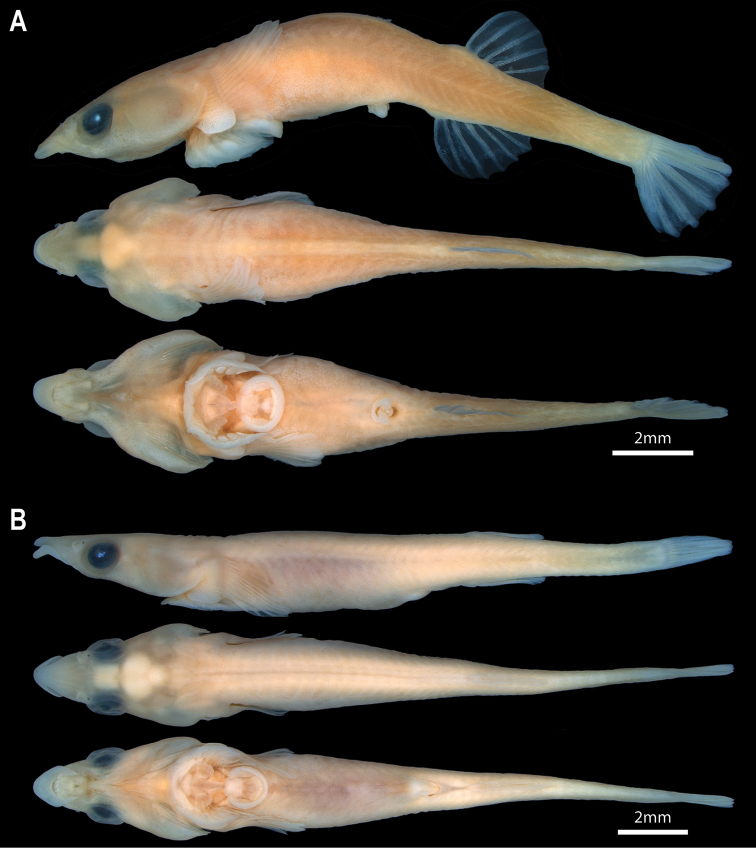
Specimens of *Barryichthys***A***B.hutchinsi*, WAM P.28981-004, holotype, male, 15.4 mm SL; Western Australia, Cottesloe Reef Platform, Perth **B***B.algicola*, WAM P.27127-016, holotype, female, 16.9 mm SL; Victoria, Jubilee Point.

**Figure 3.**
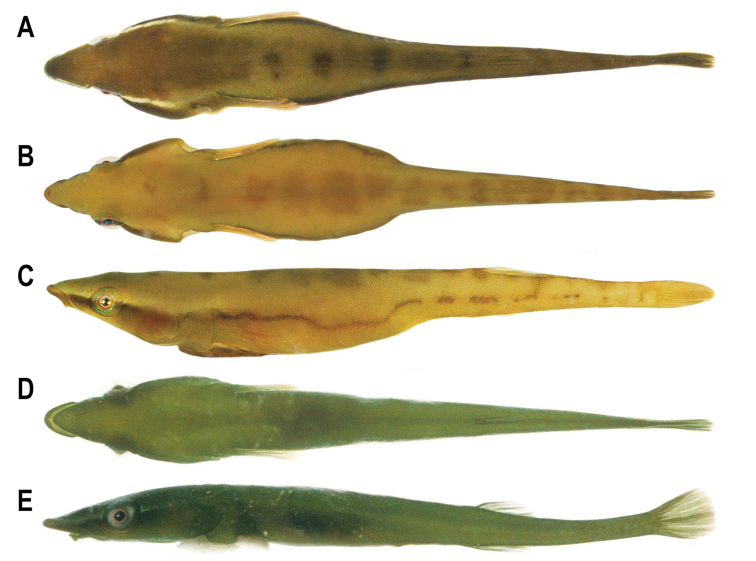
Live or freshly dead individuals of *Barryichthys***A–C***B.hutchinsi*, WAM P.28981-003, Western Australia, Cottesloe Reef Platform, Perth; male in dorsal view (**A**) female in dorsal (**B**) and lateral view (**C**). **D, E***B.algicola*, WAM P.27559-007, Tasmania, St. Helens; in dorsal (**D**) and lateral (**E**) view. Photographs by B. Hutchins.

General body shape as in Figs 2A, 3A–C. Select morphometric and meristic characters are listed in Tables 1, 2. Largest specimen examined 18.7 mm SL. Body moderately elongate, circular in cross-section anteriorly, becoming increasingly laterally compressed posteriorly. Widest point of body midway between head and dorsal-fin origin, corresponding with centre of abdominal cavity. Body width and depth tapering gradually posteriorly from widest point. Caudal peduncle thin, elongate (approximately 1/5 of SL). Head relatively large (approximately 1/3 of SL), slightly dorsoventrally compressed anteriorly, becoming increasingly circular in cross-section posteriorly. Widest point of head midway between orbit and opercular opening; wider than widest point of body. Eye large, positioned on dorsolateral surface of head; ventral margin of orbit not visible in ventral view (Fig. 5A). Snout of moderate length, triangular, narrowest anteriorly. Anterior nostril a small tubular opening (Fig. 5A). Posterior nostril surrounded by a low fleshy rim; situated along anterodorsal margin of orbit (Fig. 5A). Gill membranes united across midline, free from isthmus.

**Table 1.** Select morphometric characters obtained from the holotype and four paratypes of *Barryichthyshutchinsi* and *B.algicola*. Ranges include values from holotype.

	***Barryichthyshutchinsi* (n = 5)**				***Barryichthysalgicola* (n = 5)**			
	**Holotype**	**Range**	**Mean**	**St. Dev.**	**Holotype**	**Range**	**Mean**	**St. Dev.**
**Standard Length (SL)**	15.4	15.4–18.7			16.9	13.1–16.9		
**In % of SL**								
Head length (HL)	28.3	26.2–30.3	27.9	1.8	28.8	26.6–31.6	29.1	2
Body depth	10	9.6–11.6	10.3	0.9	8.3	7.4–8.3	7.8	0.4
Predorsal length	70.9	67.4–70.9	69.2	1.8	68.7	68.3–71.3	69.8	1.5
Preanal length	67.8	61.4–67.9	65.1	3.3	69.4	68.3–71.0	69.3	1.2
Preanus length	60.7	54.1–61.0	57.6	3.7	62.3	59.0–62.6	61.2	1.7
Anus to disc	25.1	16.6–25.1	20	4.5	26.2	22.9–26.8	25.2	1.7
Anus to anal fin	6.8	6.8–9.1	8.2	1.1	7.3	5.5–9.0	7.1	1.5
Caudal peduncle length	21.6	20.1–22.3	21.3	0.9	20.4	20.0–25.1	22.1	2.4
Caudal peduncle depth	5.8	5.2–6.2	5.7	0.4	4.9	4.1–4.9	4.5	0.4
Disc length	15	15.0–17.7	16.1	1.2	16.1	13.5–16.1	14.5	1.2
Disc width	12.6	12.6–15.0	14.1	1.3	13.2	12.1–13.4	12.8	0.6
**In % of HL**								
Head depth at orbit	31	28.2–32.5	30.4	1.9	26.1	25.3–27.3	26.3	0.8
Head width at orbit	36.9	33.8–38.2	35.9	1.9	32.7	32.7–38.2	34.9	2.4
Head width at widest point	65.8	65.3–74.9	69.1	4.5	56.6	55.2–60.9	57.1	2.6
Interorbital width	32.9	26.5–32.9	28.8	2.9	20.6	19.5–23.8	21.7	2
Snout length	25.8	24.4–25.8	24.9	0.7	30	27.7–31.1	29.5	1.4
Eye diameter	24	21.0–24.0	22.1	1.4	22.4	21.4–24.9	23.3	1.6

Mouth subterminal, small; posterior tip of upper jaw not reaching imaginary vertical line through anterior margin of orbit when mouth closed. Articulation between anguloarticular and quadrate located directly along imaginary vertical line through anterior margin of orbit. Upper lip fleshy (Fig. 5A); in dorsal view appearing uniform in thickness around entire anterior margin of snout; in lateral and ventral view upper lip appearing markedly thicker anteriorly, tapering in thickness posteriorly. Lower lip restricted to lateral margin of lower jaw only; separated along ventral midline by a fleshy pad of skin at symphysis of lower jaw. Lower lip narrower than upper lip, with poorly developed skin flap anteromedially. Fleshy pad of skin at symphysis of lower jaw bordered anterolaterally by a shallow groove; confluent posteriorly with skin of isthmus (Fig. 5A). Upper jaw longer and wider than lower jaw (Fig. 7A), creating a narrow gap between teeth of upper and lower jaw when jaws closed. Premaxilla with an outer row of 6–8 small conical teeth with slightly recurved tips, arranged along anteromedial edge, adjacent to symphysis, and a small patch of 2–4 tiny conical teeth on lingual surface posterior to teeth of outer row. Dentary with a single row of 5–6 conical teeth; anteriormost 3–4 teeth dagger-like, only slightly recurved and orientated at a 180° angle to dentary, with cusp directed anteriorly; posteriormost 2–3 teeth strongly recurved and orientated at a 90° angle to dentary, with cusp directed posterodorsally (Fig. 7A). Teeth on dentary slightly larger than largest teeth on premaxilla. Ascending process of premaxilla narrow, elongate (Fig. 7A); extending posteriorly along dorsal surface of neurocranium to a point slightly anterior to epiphyseal commissure of supraorbital lateral line canal when jaws closed. Pharyngeal jaws comprising patch of 3–4 tiny conical teeth with slightly recurved tips on pharyngobranchial 3 toothplate only (Fig. 7C); teeth absent from ceratobranchial 5 (Fig. 7B). 3–5 tiny, gnarled gill rakers along anterior and posterior edge of ceratobranchials 2–3 and anterior edge of ceratobranchial 4; ceratobranchial 1 without gill rakers (one gill raker along posterior edge of ceratobranchial 1 of left side only in one C&S specimen). Gill filaments associated with gill arches I–III only (three gill filaments of Briggs, 1955); restricted to lower (ceratobranchial) portion of gill arches only; ceratobranchial 2 and 3 with paired rows of filaments (holobranch); ceratobranchial 1 with single row (hemibranch) of 4–5 poorly developed gill filaments. Basihyal a short club-like element; capped with cartilage anteriorly (Fig. 7B). Ceratobranchials 1–4 rod-like elements; ceratobranchial 5 a short plate-like element, wider and shorter than more anterior ceratobranchial elements (Fig. 7B). Epibranchials 1–2 short rod-like elements; epibranchial 3 a club-like element, broadest anteriorly; epibranchial 4 a single splint like element (epibranchial 4 fused to epibranchial 3 on left side only in one C&S specimen; Fig. 7C). Five or six branchiostegal rays (Fig. 7D). In specimens with five, first ray articulating medially with hyoid bar along anterior ceratohyal; posterior rays articulating with hyoid bar laterally, including two along posteriormost part of anterior ceratohyal, one straddling junction between anterior and posterior ceratohyals, and one along anteriormost part of posterior ceratohyal. In specimens with six, an additional small ray without contact to hyoid bar located anterior to ray articulating with medial face of hyoid bar.

**Figure 4.**
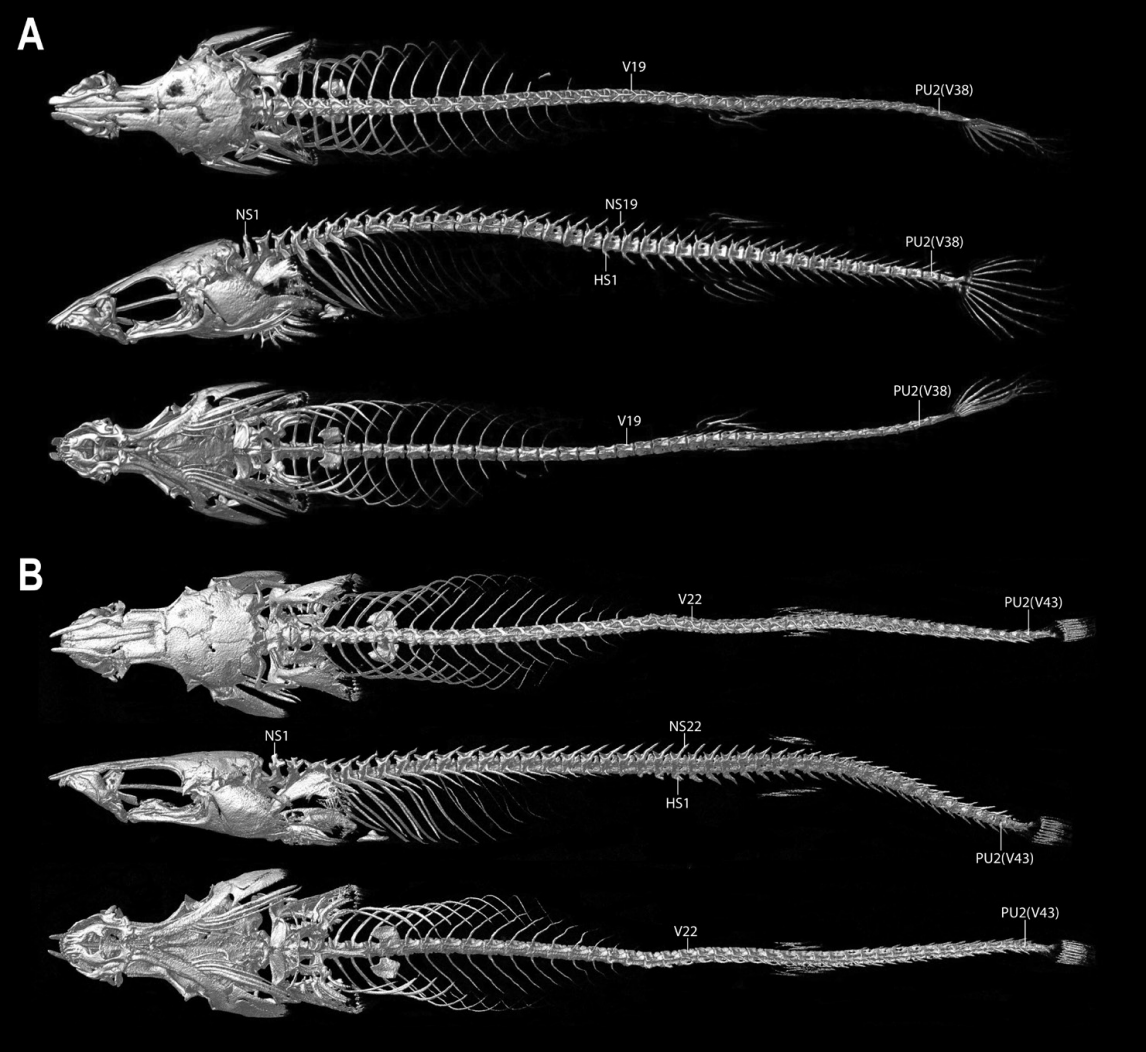
CT scanned skeleton of *Barryichthys* in dorsal, lateral and ventral view **A***B.hutchinsi*, AMS I.49000-001, 14.0 mm SL**B***B.algicola*, WAM P.27127-016, holotype, female, 16.9 mm SL. Abbreviations: HS, hemal spine; NS, neural spine; PU2, preural centrum 2; V, vertebra.

**Figure 5.**
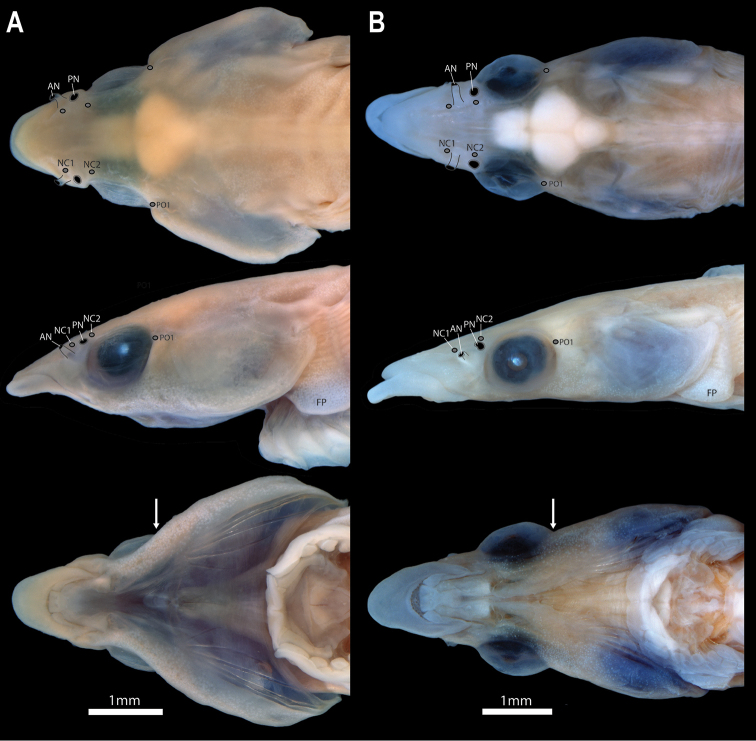
Head (in dorsal, lateral, and ventral views) in members of *Barryichthys* highlighting position of cephalic lateral line canal pores (grey circles) on head surface **A***B.hutchinsi*, WAM P.28981-004, holotype, male, 15.4 mm SL**B***B.algicola*, WAM P.27127-016, holotype, female, 16.9 mm SL. Outline of anterior and posterior nostril highlighted by grey solid line. White arrow points to posterior margin of orbit. Abbreviations: AN, anterior nostril; FP, fleshy pad at base of pectoral fin; NC1–2, nasal canal pores 1–2; PN, posterior nostril; PO1, postorbital canal pore 1.

**Figure 6.**
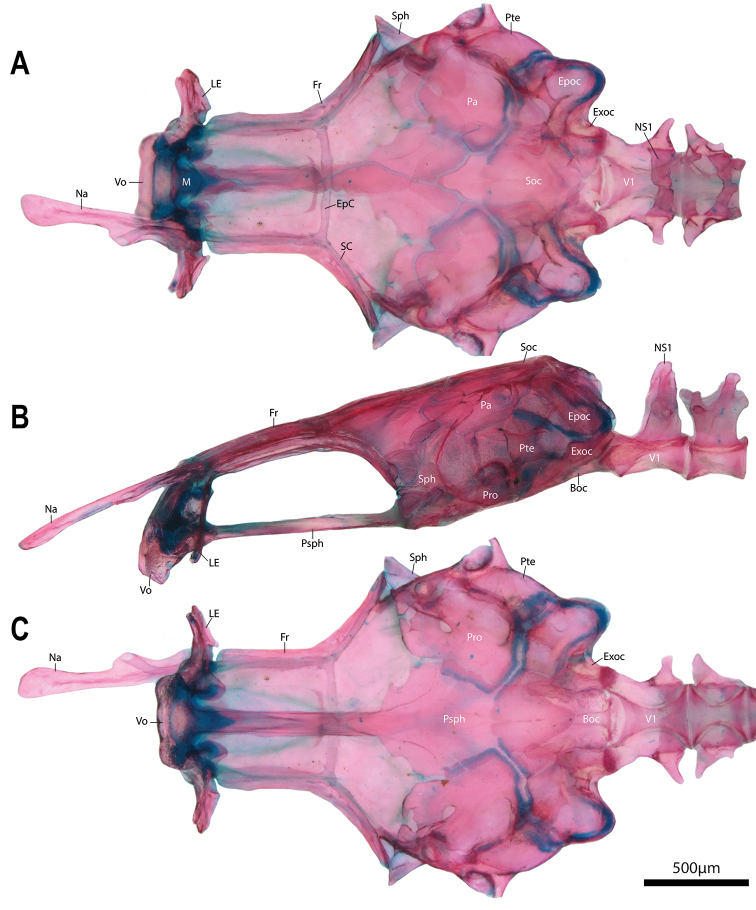
Neurocranium of *Barryichthyshutchinsi*, WAM P.34510-001, paratype, 15.5 mm SL**A** dorsal view **B** lateral view (left side) **C** ventral view. Lachrymal not shown. Nasal of right side removed. Abbreviations: Boc, basioccipital; EpC, epiphyseal commissure of supraorbital canal; Epoc, epiotic; Exoc, exoccipital; Fr, frontal; LE, lateral ethmoid; M, mesethmoid; Na, nasal; NS1, neural spine of vertebral centrum 1; Pa, parietal; Pro, prootic; Psph, parasphenoid; Pte, pterotic; SC, supraorbital canal; Soc, supraoccipital; Sph, sphenotic; V1, vertebral centrum 1; Vo, vomer.

Superficial neuromasts on surface of head not observed in material other than a pair of large superficial neuromasts housed within a pair of shallow depressions at centre of symphysial pad on lower jaw. Cephalic lateral-line system comprising supraorbital lateral-line canal only; 2 nasal pores; 1 postorbital pore. Canal pores minute; flush with surface of skin and difficult to locate. Supraorbital lateral line canals connected across midline via epiphyseal commissure (Fig. 6A). Lachrymal, a small paddle-like bone, without canal ossification, articulating with anterolateralmost point of lateral ethmoid. Nasal elongate, approximately half length of frontal, with canal ossification restricted to posteriormost part of bone adjacent to olfactory capsule. Nasal bones extending far anterior to ethmoid region of neurocranium over dorsal surface of upper jaw; terminating anterior to anteriormost point of upper jaw (Fig. 4A). Parasphenoid widest posteriorly ventral to occipital region of neurocranium; tapering anteriorly and abruptly to a narrow strut of bone along ventral midline of neurocranium (Fig. 6).

Dorsal-fin rays 4 or 5(*). Anal-fin rays 4, 5 or 6(*). All dorsal- and anal-fin rays unbranched and segmented; each in serial association with a narrow, rod-like pterygiophore, comprising proximal-middle radial only. Principal caudal-fin rays 4+4, dorsal procurrent rays 1 or 2, ventral procurrent rays 1 or 2. Principal caudal-fin rays and posteriormost dorsal and ventral procurrent rays unbranched and segmented; anteriormost dorsal and ventral procurrent ray unsegmented. Pectoral-fin rays 15 or 17; uppermost ray typically a tiny splint-like element comprised of a single hemitrichium; present on right side only in one C&S specimen (WAM P.34510-001). Lowermost 4–5 pectoral-fin rays more heavily ossified and approximately half length of upper rays, with foreshortened segments in each hemitrichium (sensu Lundberg & Marsh 1976) (Fig. 8). Remaining pectoral-fin rays (uppermost 10–12 rays) poorly ossified, without segmentation of hemitrichia (Fig. 8). Pelvic-fin rays I.4. Distal tip of spinous pelvic-fin ray narrow; strongly bifurcated proximally, embracing a small circular cartilaginous pelvic-radial cartilage. Pelvic-fin rays 1–3 increasing in length and width posteriorly. Caudal fin marginally truncate, tips of principal caudal-fin rays extended slightly beyond fin margin. Caudal-fin skeleton comprised of narrow upper and lower hypural plates (Fig. 10B); lower hypural plate with short antero- and posteroventral processes along ventral surface; tip of posteroventral process capped with cartilage. Epural a narrow, roughly triangular element, wider posteriorly than anteriorly, with broad cartilaginous posterodorsal margin; parhypural cartilage a small irregular element located at tip of posteroventral process of lower hypural plate (Fig. 10B). Dorsal-fin origin opposite anal-fin origin (Figs 2A, 4A). First dorsal-fin pterygiophore inserted between neural spines of vertebrae 20/21 or 21/22. First anal-fin pterygiophore inserted between hemal spines of vertebrae 19/20 or 20/21. Proximal-middle radials of dorsal- and anal-fin pterygiophores rod-like, without cup-like anterior process (Fig. 10A). Total number of vertebrae 38 or 39, consisting of 17 abdominal vertebrae and 21 or 22 caudal vertebrae (Fig. 4A). Ribs 11 or 12 associated with vertebrae 3–13/14. Epicentrals 14 or 15, associated with vertebrae 3–16/17.

Adhesive disc small (15–18% of SL), double (Fig. 9A); outer margin of disc smooth. Outline of anterior margin of disc slightly irregular, concave at midline. Posterior margin of smaller inner disc bordered by narrow flap of dense skin which has rolled inward in majority of specimens, concealing outer papillae of disc region B. Disc region A without papillae at centre; inner margin with single row of elongate papillae, transitioning to smaller papillae with circular or cuboid margins posterolaterally over ventral surface of pectoral-fin rays. Apapillate region of disc region A equal in width or slightly narrower than width of smaller inner disc. Disc region B with 2 transverse rows of papillae, comprised largely of elongate papillae with few smaller papillae with circular or cuboid margins scattered between elongate papillae. Disc region C covered in a thick pad of skin; apapillate. Disc region D with an irregular U-shaped papilla (Fig. 9A) or 2–3 circular to cuboid papillae at centre (Fig. 1A). Smaller inner disc connected to larger outer disc anteriorly via a narrow frenum of thick skin along ventral midline. Skin of frenum confluent with posterior margin of disc region D; lateral margins of frenum smooth to weakly crenate (Fig. 9A). Dorsal postcleithrum a poorly ossified sheet of bone with ~20 long, poorly ossified fimbrae along posterior margin (Fig. 9C). Medial edge of dorsal postcleithrum with a short peg-like strut of bone, directed towards ventral midline. Ventral postcleithrum well ossified, irregular in shape; approximately half size of dorsal postcleithrum (Fig. 9C). Posterior margin of ventral postcleithrum smooth, without fimbrae. Anteromedial edge of ventral postcleithrum with a concave facet that articulates with a dense pad of connective tissue located at posterior tip of basipterygium (Fig. 9C). Skin associated with last pelvic-fin ray attaching to base of pectoral fin opposite 4^th^–5^th^ lowermost pectoral-fin rays. Skin over base of ventral pectoral-fin rays and lower half of shoulder girdle swollen and creating an obvious skin pad; epidermis of pad with a dense aggregation of club cells, giving skin pad a whitish appearance in preserved specimens (Fig. 8A). Pectoral radials with well-developed bony struts along ventral (pectoral radial 1), dorsal (pectoral radial 4), or both ventral and dorsal margins (pectoral radials 2 and 3) that interdigitate with struts borne on element(s) directly above and/or below (Fig. 8B, C).

**Figure 7.**
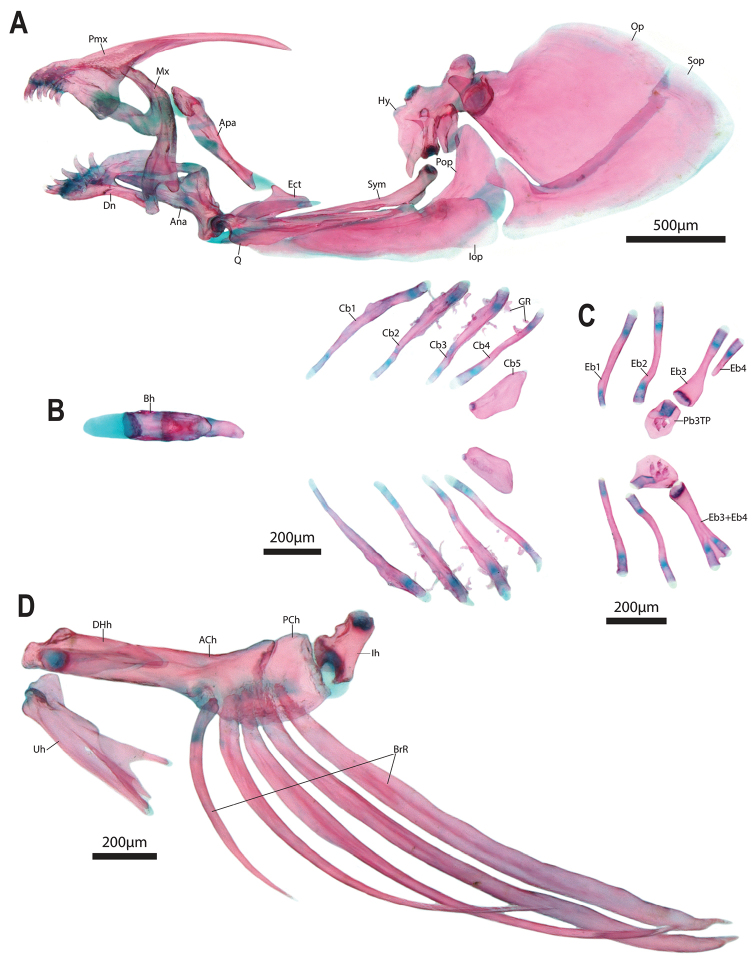
Viscerocranium of *Barryichthyshutchinsi*, WAM P.34510-001, paratype, 15.5 mm SL**A** hyopalatine arch and opercular series, right side in lateral view (image reversed) **B** lower gill-arch elements in dorsal view, gill filaments removed **C** upper gill-arch elements in ventral view **D** hyoid bar, right side in medial view and urohyal. Abbreviations: ACh, anterior ceratohyal; Ana, anguloarticular; Apa, autopalatine; Bh, basihyal; BrR, branchiostegal rays; Cb1-5, ceratobranchial 1–5; DHh, dorsal hypohyal; Dn, dentary; EB1–4, epibranchials 1–4; EB3+4, compound element comprising EB3 and EB4; Ect, ectopterygoid; GR, gill raker; Hy, hyomandibular; Iop, interopercle; Mx, maxilla; Op, opercle; Pb3TP, pharyngobranchial 3 toothplate; Pop, preopercle; Q, quadrate; Sop, subopercle; Sym, symplectic; Uh, urohyal.

#### Colouration.

In alcohol, head and body background colour uniformly pale cream to yellow (Fig. 2A). In life, head and body background colour golden-yellow to olive-brown (Fig. 3A–C). Dorsal midline with variable number (10–14) of irregularly shaped light to dark brown markings; markings largest dorsal to centre of body, becoming smaller anterior or posterior to this point. Body side with a series of light to dark brown elongate markings forming an incomplete or complete horizontal stripe. Horizontal light to dark brown stripe along side of body continuing on side of head, through lower half of eye, to snout. Dorsal margin of light to dark brown stripe on head bordered by a lighter stripe, ranging from light yellow to white. Lighter stripe more pronounced in males. Iris red to orange. Fins uniform in colour without markings; colour matching body background colour.

#### Sexual dimorphism.

External sexual dimorphism largely restricted to urogenital papilla. Urogenital papilla of male with a blunt tip, located within a deep groove posterior to the anus and flanked anterolaterally by a pair of swollen skin folds, termed here accessory folds. Each accessory fold is roughly triangular in shape and appears to be confluent anteromedially with the heavily plicate skin surrounding the anus (Fig. 11A, B). Urogenital papilla of female with a needle-like tip, located along the dorsal surface of a robust tube-like structure which also bears the anus (Fig. 11C, D). This entire structure is accommodated within a deep pocket anterior to the anal-fin origin. In several specimens, the posteriormost tip of this structure is located within the pocket, suggesting some degree of mobility.

#### Eggs.

A female of 14.2 mm SL from WAM P.34510-001 contained ca. 20 mature eggs (ca. 10 within each ovary) of ca. 0.3–0.6 mm diameter. The largest eggs in each ovary exhibited a dark orange cap that may represent an “attachment apparatus” at the animal pole as described from the eggs of three species of European gobiesocid by Breining and Britz (2000).

#### Distribution.

Known presently only from two close sites in Western Australia (Cottesloe Reef and Trigg Reef platforms, Perth) and two sites in South Australia (Vivionne Bay and Victor Harbor) (Fig. 12). At the type locality (Cottesloe Reef platform), *B.hutchinsi* was collected from dense mats of macroalgae attached to rocky substrate in water up to 1 meter depth.

#### Etymology.

Named for Barry Hutchins, who discovered the new species. A noun in the genitive.

#### Remarks.

Hutchins (2008: 725) illustrated a specimen of *Barryichthyshutchinsi* from Western Australia, likely from the type locality at Cottesloe Reef platform (Perth). Specimens from South Australia (AMS I.20171-012, AMS I.49000-001) exhibit vertebral counts within the range of *B.hutchinsi* and are referred to this species. These specimens have been excluded from the type series but data obtained from these specimens has contributed to the description above.

**Figure 8.**
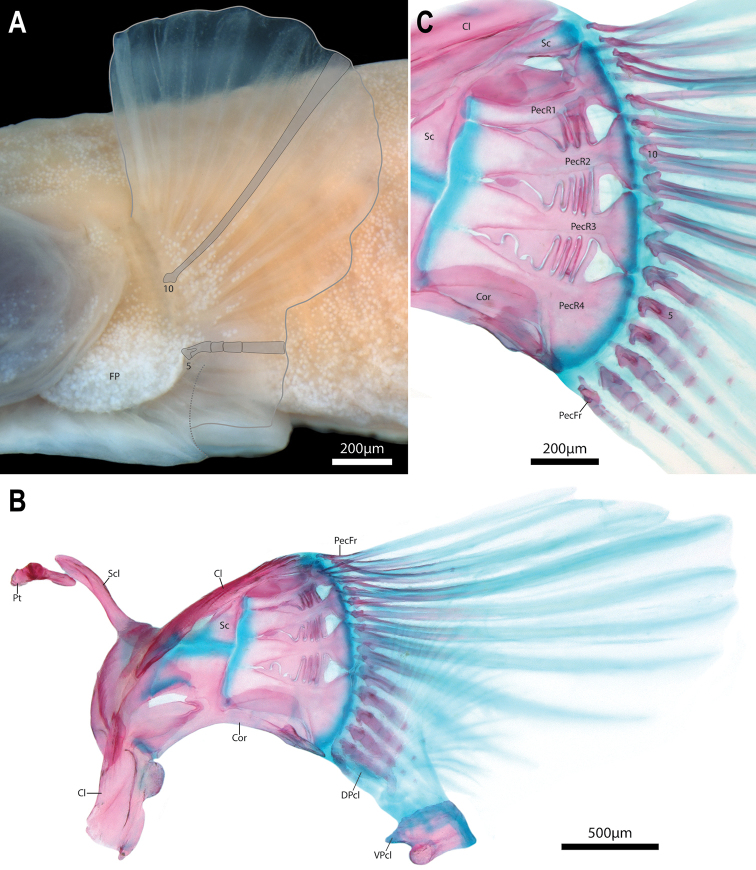
Pectoral fin and pectoral-fin girdle of *Barryichthyshutchinsi***A** pectoral fin, left side in lateral view, WAM P.28981-004, holotype, male, 15.4 mm SL. Outline of fin margin highlighted by thin grey line. Schematic representation of 5^th^ and 10^th^ pectoral-fin rays (counted from ventral to dorsal) overlay rays **B** pectoral-fin girdle, right side in medial view, WAM P.34510-001, paratype, 15.5 mm SL**C** close-up of area of articulation between pectoral-fin rays and girdle, right side in medial view (image reversed; same specimen as in B). Postcleithra removed. Abbreviations: Cl, cleithrum; Cor, coracoid; DPcL, dorsal postcleithrum; PecR1–4, pectoral radial 1–4; PecFR, pectoral-fin ray; Pt, posttemporal; Sc, scapula; Scl, supracleithrum; VPcl, ventral postcleithrum; 5, 10, 5^th^ and 10^th^ pectoral-fin ray (counted from ventral to dorsal).

**Figure 9.**
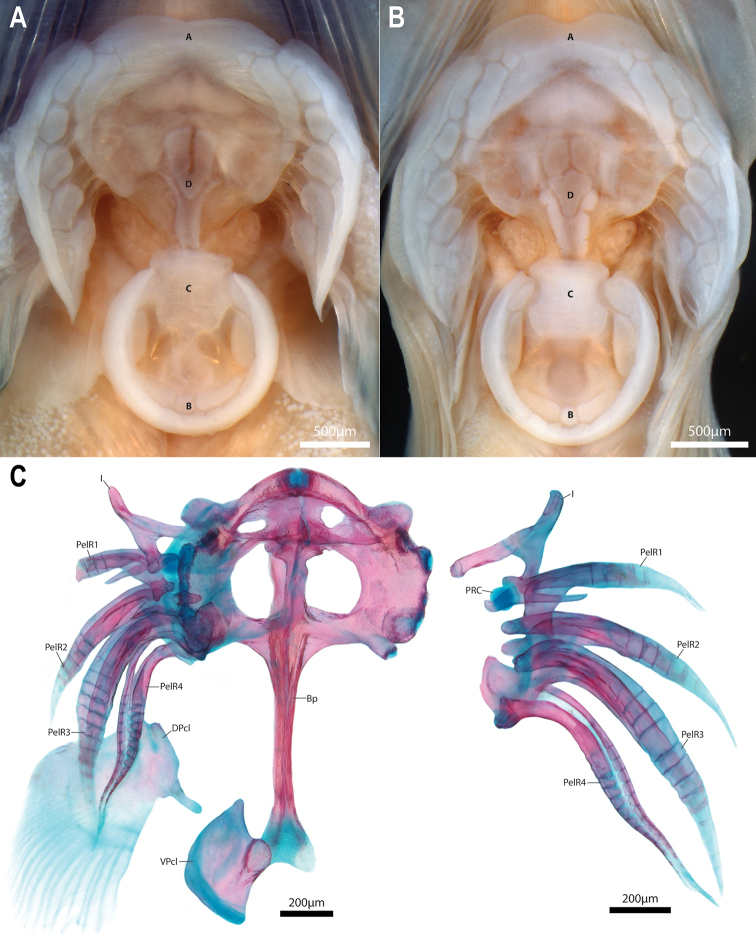
Surface features (**A, B**) and internal supporting skeleton (**C, D**) of the adhesive disc of *Barryichthys***A** adhesive disc of *Barryichthyshutchinsi* (WAM P.28981-004, holotype, male, 15.4 mm SL), ventral view (anterior to top of page) **B** adhesive disc of *B.algicola* (WAM P.27127-016, holotype, female, 16.9 mm SL), ventral view (anterior to top of page) **C** adhesive disc supporting skeleton, including elements of the pelvic and pectoral-fin girdle of *B.hutchinsi* (WAM P.34510-001, paratype, 15.5 mm SL), ventral view (anterior to top of page). Postcleithra and pelvic-fin rays of the right side removed (image reversed) **D** pelvic-fin spine and rays of right side of *B.hutchinsi* (same specimen as in C), dorsal view (anterior to top of page). Abbreviations: A, disc region A; B, disc region B; Bp, basipterygium; C, disc region C; D, disc region D; DPcL, dorsal postcleithrum; I, pelvic-fin spine; PelR1–4, pelvic-fin rays 1–4; PRC, pelvic-radial cartilage; VPcL, ventral postcleithrum.

### Barryichthys algicola sp. nov.

Taxon classificationAnimaliaGobiesociformesGobiesocidae

http://zoobank.org/ACAC214A-2D61-40B4-82E3-DBB5C55474A2

Figures 2B , 3D, E , 4B , 5B , 6B Common name: Green rat clingfish

Genus B sp., Hutchins 1994a: 310

#### Holotype.

WAM P.27127-016, female, 16.8 mm SL; Victoria, Jubilee Point, Sorrento (38°20'00"S, 144°45'00"E), 3 March 1981, J.B. Hutchins, CT scan https://doi.org/10.17602/M2/M78489.

#### Paratypes.

*New South Wales*: AMS I.137167-002, 1, 14.2 mm SL; Cape Banks, Botany Bay (34°00'00.0"S, 151°15'00.0"E), 01 March 1992–06 July 1993, N Gallahar. *Victoria*: WAM P.27127-001, 2, 16.0–21.0 mm SL; Same as holotype. *Tasmania*: AMS I.17555-002, 3, 15.5–15.7 mm SL; The Gardens, north of Binalong Bay (41°14'21.3"S, 148°17'35.8"E), D. Hoese & W. Ivanstoff. – AMS I.17576-012, 1, 19.0 mm SL; The Gardens, north of Binalong Bay (41°14'21.3"S, 148°17'35.8"E), D. Hoese & W. Ivanstoff. – AMS I.46787-001, 1, 15.6 mm SL; Coles Bay (42°07'28.0"S, 148°16'54.0"E), H. Lloyd. – WAM P.27572-004, 2, 10.0–13.0 mm SL; West Point, Marrawah (40°55'00"S, 144°42'00"E), 13 March 1982, J.B. Hutchins. – WAM P.27576-003, 1, 14.0 mm SL; north side of Granville Harbour (41°49'00"S, 145°01'00"E), 18 March 1982, J.B. Hutchins. – WAM P.27559-007, 10 (2C&S), 9.0–12.0 mm SL; St. Helens Point (41°16'00"S, 148°22'00"E), 25 February 1982, J.B. Hutchins.

#### Diagnosis.

*Barryichthysalgicola* is distinguished from *B.hutchinsi* by a longer, narrower body (body depth at dorsal-fin origin 7–8% SL vs. 10–11% SL), a more slender head (head width at widest point 55–61% HL vs. 66–75%; depth at orbit 27–29% HL vs. 30–32%; interorbital width 20–24% vs. 27–33% HL), the entire ventral margin of the orbit visible in ventral view (vs. ventral margin of orbit obscured by cheek in ventral view), by having a longer abdominal region with more vertebrae (abdominal vertebrae 21 vs. 17) and more ribs (15 vs. 11–12), a higher number of epicentrals (18–19 vs. 14–15), and a higher total number of vertebrae (42–44 vs. 38–39), and by features of live colour pattern, including body background colour green (vs. golden-yellow to olive-brown) without darker markings along dorsal midline or body side (vs. dorsal midline and lateral body side with darker markings).

#### Description.

General body shape as in Figs 2B, 3D–E. Select morphometric and meristic characters are listed in Tables 1, 2. As described for *B.hutchinsi* except for the following differences. Largest specimen examined 21.0 mm SL. Head narrow; widest point of head only slightly wider than widest part of body. Entire ventral margin of orbit visible in ventral view (Fig. 5B). Dorsal-fin rays 5 or 6. Anal-fin rays 6. Pectoral-fin rays 17. First dorsal-fin pterygiophore inserted between neural spines of vertebrae 23/24. First anal-fin pterygiophore inserted between hemal spines of vertebrae 20/21 or 22/23. Total number of vertebrae 42, 43(*) or 44, consisting of 21 abdominal vertebrae and 21, 22(*) or 23 caudal vertebrae (Fig. 4B). Ribs 15, associated with vertebrae 3–17. Epicentrals 18 or 19, associated with vertebrae 3–20/21.

**Table 2.** Total number of vertebrae in specimens of *Barryichthys*. Number obtained from holotype indicated with an asterisk.

**Species**	**N**	**Number of Vertebrae**						
		38	39	40	41	42	43	44
* B. hutchinsi *	8	3	5*	–	–	–	–	–
* B. algicola *	11	–	–	–	–	3	4*	4

#### Sexual Dimorphism.

As described for *B.hutchinsi*.

#### Eggs.

A female of 17.2 mm SL from WAM P.27127-001 contained multiple mature eggs (number not counted) in the right ovary. A single excised egg (ca. 0.6 mm in diameter) exhibited a dark orange cap that may represent an “attachment apparatus” at the animal pole as described from the eggs of three species of European gobiesocid by Breining and Britz (2000).

#### Colouration.

In alcohol, head and body background colour pale cream (Fig. 2B). In life, head and body uniformly green (Fig. 3D, E). A lighter green stripe on side of head, extending from tip of snout to upper part of gill opening, passing through eye. Iris orange. Pectoral fin light green. Dorsal- and anal-fin rays green; fin membranes hyaline. Caudal-fin rays green; fin membranes light green.

#### Distribution.

Known presently from multiple sites along the northern and northeastern coast of Tasmania, and two sites along the coast of mainland Australia, including Jubilee Point (Victoria; type locality) and Botany Bay (New South Wales) (Fig. 12). The majority of specimens have been collected from subtidal fields of macroalgae, 0–2 meters depth.

#### Etymology.

Neologism combining the Latin *alga* and *colare*, who inhabits the algae, in reference to the habitat preference of the new species. A noun in apposition.

#### Remarks.

The specimen of “Rat clingfish” illustrated in Hutchins (1994a: 310, fig. 273) represents *Barryichthysalgicola*. An elongate gobiesocid larva (AMS I.48745-008) collected along the coast of New South Wales have been tentatively identified as *B.algicola* (T. Miskiewicz, pers. comm.)

**Figure 10.**
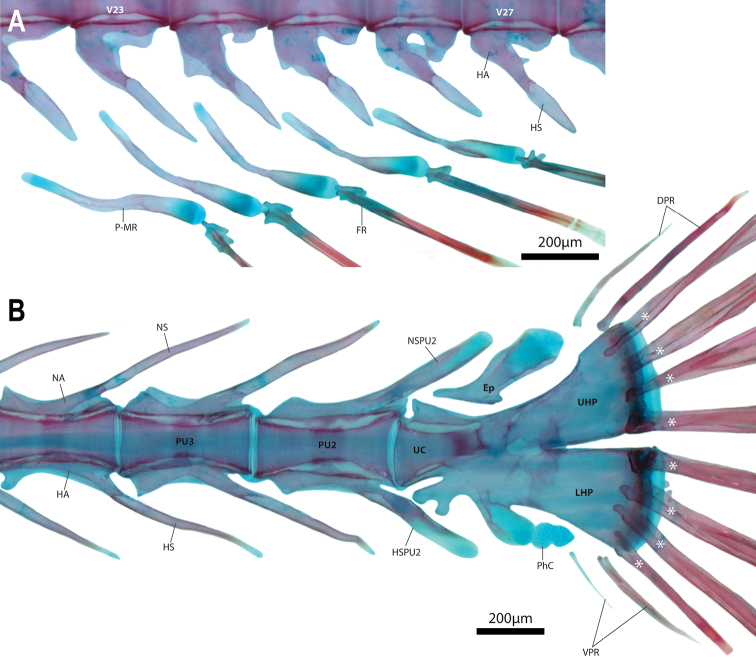
Anal- and caudal-fin skeleton of *Barryichthyshutchinsi*, WAM P.34510-001, paratype, 15.5 mm SL**A** anal-fin skeleton, left side in lateral view **B** caudal-fin skeleton, left side in lateral view. Principal caudal-fin rays are labelled with an asterisk (*). Abbreviations: DPR, dorsal procurrent rays; Ep, epural; FR, fin ray; HA, hemal arch; HS, hemal spine; HSPU2, hemal spine of preural centrum 2; LHP, lower hypural plate; NA, neural arch; NS, neural spine; NSPU2, neural spine of preural centrum 2; PhC, parhypural cartilage; P-MR, proximal-middle radial; PU2-3, preural centrum 2, 3; UC, ural centrum; UHP, upper hypural plate; VPR, ventral procurrent rays.

**Figure 11.**
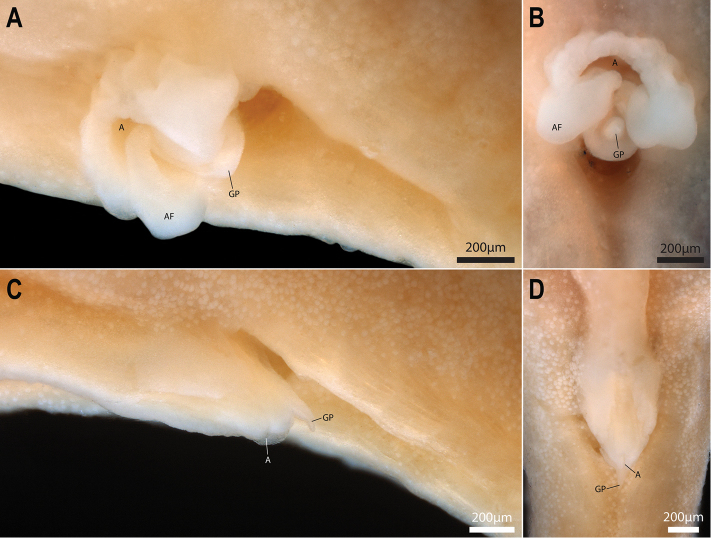
Genital papilla of *Barryichthyshutchinsi***A**WAM P.28981-004, holotype, male, 15.4 mm SL, oblique lateral view **B** same specimen as in A, ventral view, anterior to top of page **C**WAM P.28981-003, paratype, female, 16.9 mm SL, oblique lateral view **D** same specimen as in D, ventral view, anterior to top of page. Abbreviations: A, anus; AF, accessory folds; GP, genital papilla.

**Figure 12.**
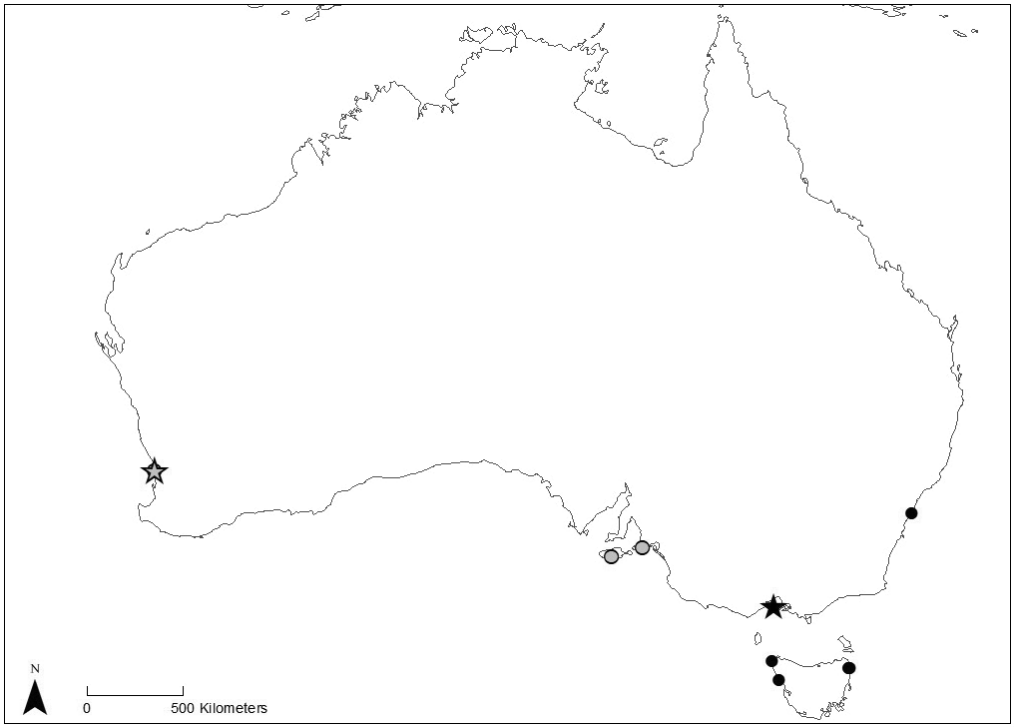
Distribution of material of *Barryichthyshutchinsi* (grey symbols) and *B.algicola* (black symbols) used in this study. Stars indicate type localities.

## Discussion

Specimens of *Barryichthys* have been known since at least the mid-1980s and referred to either as an undescribed genus (Last et al. 1983), as “Genus B” (Hutchins 1994a, 2008), or simply as “rat clingfish” (Hutchins 1991b). Hutchins (2008) considered his Genus B (here *Barryichthys*) to be monotypic, with a single undescribed species exhibiting a disjunct distribution along the southern coast of Australia, including Western Australia to the west and Victoria and Tasmania to the east. Our examination of material in museum collections has resulted in additional material of *Barryichthys* not known to Hutchins (2008) and from additional localities outside of the suspected range of the genus, including those in South Australia (Kangaroo Island and Victor Harbor) and New South Wales (Botany Bay). As we have shown herein, this material comprises two distinct species, with non-overlapping distributions along the southern coast, including the more western distributed *B.hutchinsi*, with specimens known from Western Australia and South Australia, and the more eastern distributed *B.algicola*, with specimens known from New South Wales, Victoria, and Tasmania. This disjunct distribution of *Barryichthys* is another example of numerous geminate species divided by the Bass Strait (see Moore 2012). The most parsimonious explanation for the presence of so many geminate pairs across a wide range of taxa is repeated vicariant isolations by an ephemeral biogeographic barrier in the form of a landbridge between southern Australia and Tasmania during historic glacial cycles (Hutchins 1994b; Burridge 2000; Waters et al. 2004; Moore and Chaplin 2014). Species endemic to the west of the Bass Strait may have distributions restricted to the south-west corner or be widespread across southern Western Australia and South Australia (Hutchins 1994b). Based on this and our morphological evidence, we believe the South Australian specimens included here do represent *B.hutchinsi*, but further work on specimens from this region may be warranted.

The two species of *Barryichthys* are similar in overall appearance but differ in aspects of head shape, number of vertebrae and aspects of live colouration. The most obvious external difference between *B.hutchinsi* and *B.algicola* relates to the eye, the entire ventral margin of which is visible in ventral view in *B.algicola* (Fig. 5B) but not in *B.hutchinsi*, in which only the lateralmost part of the eye is visible in ventral view with the ventral eye margin obscured by the cheek (Fig. 5A). *Barryichthyshutchinsi* exhibits fewer vertebrae than *B.algicola* (38–39 vs. 42–44; Table 2) and these differences appear to be related to differences in the length of the abdominal region of the vertebral column, which is comprised of fewer vertebrae in *B.hutchinsi* (17) than in *B.algicola* (21). *Barryichthyshutchinsi* also exhibits fewer epicentrals than *B.algicola* (14–15 vs. 18–19) and there are also fewer ribs surrounding the abdominal cavity of *B.hutchinsi* compared to that of *B.algicola* (11–12 vs. 15). In contrast, the number of caudal vertebrae is similar in both species (21–22 in *B.hutchinsi* vs. 21–23 in *B.algicola*). In life, *B.hutchinsi* exhibits an overall golden-yellow to olive-brown body background colour combined with a variable number of irregular shaped light to dark brown markings along the dorsal and lateral body surface whereas the body background colour of *B.algicola* is uniform green in life and without obvious markings. Photographs of live or freshly dead specimens of *B.hutchinsi* from the type locality in Western Australia reveal the presence of an obvious light yellow to white stripe along the side of the head that is not obvious in photographs of live or freshly dead specimens of *B.algicola*. This stripe may represent an additional diagnostic character between the two species but more observations are needed to confirm this, including information on live colouration of individuals of *B.hutchinsi* from South Australia.

### Comparisons with other genera

Hutchins (1994a, 2008) noted that specimens of *Barryichthys* (referred to as Genus B) are often found with members of *Parvicrepis* and several of the specimens of *Barryichthys* that we examined as part of this study were originally identified as *Parvicrepisparvipinnis* (Waite, 1906) or *Parvicrepis* sp. As previously pointed out by Hutchins (1994a, 2008), the two genera can be distinguished by features of the adhesive disc (see Fig. 1), including margin of disc region B smooth in *Barryichthys* vs. surrounded by small fleshy tabs in *Parvicrepis*, papillae absent from disc region C in *Barryichthys* vs. present in *Parvicrepis*, and disc region D with a patch of 2–3 larger circular-cuboid papillae (Fig. 1A, 9B) or a single, large, irregular-shaped papilla (Fig. 9A) in *Barryichthys* vs. a patch of 7–10 smaller circular-cuboid papillae in *Parvicrepis*. Notably, the adhesive disc in species of *Barryichthys* exhibits two distinct types of papillae (Fig. 1A), including a smaller, more ‘typical’ papilla with a circular-cuboid margin; and a larger, more elongate papilla that is approximately three to four times larger than the former. Both types of papillae exhibit smooth surfaces without obvious grooves and we suspect (though cannot confirm based on available material) that each larger, elongate papilla, develops as a single unit (i.e., the larger papillae are not the result of ontogenetic fusion between multiple smaller papillae). Small papillae with circular-cuboid margins are almost ubiquitous across the disc-bearing gobiesocids (i.e., all genera excluding *Alabes* Cloquet, 1816), with few exceptions (papillae are reported to be absent only in *Gymnoscyphusascitus* Bohlke & Robins, 1970; Bohlke and Robins 1970, Conway and Prestridge 2011), and likely represent the plesiomorphic condition at the level of the Gobiesocidae. The elongate papillae present in disc regions A and B of *Barryichthys* are unique to this genus among the disc-bearing gobiesocids and interpreted as an apomorphic condition.

In addition to features of the adhesive disc, *Barryichthys* is further distinguished from the superficially similar looking *Parvicrepis* by the presence (vs. absence) of a well-developed fleshy pad at the base of the lower pectoral-fin rays (Fig. 2, 8B), and features of the snout and jaws, including upper lip thicker at centre than at lateral margins in ventral view in *Barryichthys* vs. upper lip of uniform thickness in both dorsal and ventral view in *Parvicrepis*, and upper jaw longer than lower jaw in *Barryichthys* vs. upper and lower jaws equal in length or lower jaw only slight shorter than the upper in *Parvicrepis*.

A suite of absences and reductions also serve to distinguish *Barryichthys* from *Parvicrepis* (and also the majority of other gobiesocids), including: (1) lachrymal lateral line canal absent in *Barryichthys* vs. lachrymal lateral line canal present with two openings in *Parvicrepis* (canal absent or present with 2 or 3 openings in other gobiesocids); (2) anterior half of parasphenoid reduced to a thin strut of bone in *Barryichthys* vs. anterior half of parasphenoid broad in *Parvicrepis* (and the majority of other gobiesocids); (3) lower pharyngeal jaw teeth absent in *Barryichthys* vs. lower pharyngeal jaw teeth present, comprising a single row of 4–5 teeth on ceratobranchial 5 in *Parvicrepis* (present or absent in other gobiesocids); (4) hypobranchial and basibranchial elements (including cartilages) absent in *Barryichthys* vs. hypobranchial elements 1–3 and basibranchial cartilages 3–4 present in *Parvicrepis* (elements highly variable across Gobiesocidae; see below); and (5) uppermost 10–12 pectoral-fin rays each comprising a pair of poorly ossified and unsegmented hemitrichia in *Barryichthys* vs. hemitrichia of uppermost pectoral-fin rays comprising multiple segments in *Parvicrepis* (and other gobiesocids). The first three of these aforementioned reductions are not unique to *Barryichthys* amongst gobiesocids. For example, lachrymal sensory pores (and potentially also the lachrymal lateral line canal) are absent in *Lepadichthysakiko* Allen and Erdmann, 2012 (Fujiwara and Motomura 2018), the anterior part of the parasphenoid is reduced to a thin strut of bone in *Alabes* (Springer and Fraser 1976: Fig. 1c) and *Diademichthys* Pfaff, 1942 (Hayashi et al. 1986), and lower pharyngeal jaw teeth are absent in *Discotrema* Briggs, 1976 and *Lepadichthyslineatus* Briggs, 1966 (Conway pers. obs.). There is considerable variation in the composition of the ventral gill arch elements across the Gobiesocidae, particularly the basibranchial and hypobranchial elements (Springer and Fraser 1976). The two anteriormost basibranchial cartilages are invariably absent in all members of the Gobiesocidae (Springer and Fraser 1976) and the two posterior elements (referred to as basibranchial 3 and 4 cartilages by Springer and Fraser 1976) are variably absent (e.g., only one [typically the third] may be absent or rarely both). The most common condition of the hypobranchial elements in gobiesocids is for all three to be present and ossified (e.g., see Springer and Fraser 1976: Fig. 4b) although other conditions exist, including one in which all three hypobranchial cartilages are present but only the first is ossified as hypobranchial 1 (e.g., see Conway et al. 2018: Fig. 8C) and another in which the first element is absent and the second and third elements are present and ossified as hypobranchials 2 and 3, respectively (as in *Alabes*; see Springer and Fraser 1976: Fig. 8a). The combined absence of hypobranchial and basibranchial elements in *Barryichthys* is, as far as we are aware, unique amongst gobiesocids and is reminiscent of the extreme condition found in some members of the Anguilliforms in which all hypobranchial and basibranchial elements are absent (e.g., *Gymnothorax* Bloch, 1795 or *Cyema* Günther, 1878; Nelson 1966). The poorly ossified uppermost 10–12 pectoral-fin rays that are each comprised of a pair of unsegmented hemitrichia is another unique character of *Barryichthys* amongst gobiesocid fishes in which the hemitrichia of the pectoral-fin rays are invariably segmented in the adult stage, as is the case in most teleosts (Lundberg and Marsh 1976; Marsh 1977; Grandel and Schulte-Merker 1998).

Despite the long list of differences between *Barryichthys* and *Parvicrepis*, the two genera share a number of characteristics, including: (1) the absence of the preoperculo-mandibular lateral line canal; (2) the absence of the otic lateral line canal (=postorbital canal of Shiogaki and Dotsu 1983), with only a single sensory canal pore (PO1) posterior to orbit; (3) the absence of papillae from the centre of disc region A; (4) 4+4 principal caudal-fin rays; (5) lower 5–6 pectoral-fin rays notably shorter than upper rays, with segments foreshortened; (6) first gill arch with a few (4–5) gill filaments arranged as a hemibranch; (7) absence of filaments on the 4^th^ gill arch; (8) a double adhesive disc; and (9) gill membranes united and free from isthmus. The question of whether this long list of shared characters between *Barryichthys* and *Parvicrepis* is the result of shared common ancestry or the result of convergence is a difficult one to answer and must await the outcome of phylogenetic analysis (which is beyond the scope of this paper). The majority of the characters listed above are reductive in nature and may not be useful for grouping small-bodied taxa because the shared absences may be linked to independent cases of reduction (e.g., see Britz et al. 2014). The fact that many of these reductive characters are common to many small-bodied gobiesocid fishes (especially reductions in the cephalic lateral line canal system; Shiogaki and Dotsu 1983) lends some weight to this argument.

### Miniaturisation

Miniaturisation, the evolution of tiny adult body size, is a common phenomenon in animal taxa, especially in non-amniote vertebrates (Hanken and Wake 1993), with many notable examples from teleost fishes (e.g., Winterbottom and Emery 1981; Springer 1983; Iwata et al. 2001; Watson and Walker 2004; Kottelat et al. 2006; Britz et al. 2009). In their review of miniaturisation, Hanken and Wake (1993) noted that it is common for miniature taxa to exhibit higher numbers of morphological reductions and greater levels of morphological variability (e.g., asymmetry) in comparison to larger-bodied close relatives. They also noted that miniature taxa typically exhibited morphological novelties compared to larger-bodied close relatives and considered the evolution of morphological novelty a common consequence of the miniaturisation process (Hanken and Wake 1993). In ichthyological circles, miniature taxa are typically identified as those that mature at ≤ 20 mm SL or, when information on size at maturity is not available, are not known to exceed a maximum SL of 26 mm (following Weitzman and Vari 1988). Using these criteria, ichthyologists have identified several hundred species of miniature freshwater fishes, mostly from temperate and tropical regions (e.g., Weitzman and Vari 1988; Kottelat and Vidthayanon 1993; Conway and Moritz 2006; Bennett and Conway 2010; Toledo-Piza et al. 2014). We expect that similar numbers of marine fishes would also be identified as miniature using these criteria, if or when they are applied in the same way to the marine ichthyofauna.

Rüber et al. (2007) and Britz and Conway (2009) identified two distinct types of miniature taxa amongst cyprinid fishes, comprising: (1) proportioned dwarfs, representing scaled down replicas of closer relatives, with few reductions and few or no morphological novelties compared to their close relatives; and (2) developmentally truncated (= progenetic) miniatures, resembling earlier developmental stages of closer relatives, with high numbers of reductions and many morphological novelties. Based on these earlier observations, Britz and Conway (2016) concluded that the evolution of morphological novelty in miniature cyprinid fishes may be tied to extreme developmental truncation, which may work to release developmentally truncated taxa from the evolutionary constraints imposed on larger bodied close relatives and facilitate the evolution of novel structures. Though there is compelling evidence from miniature cyprinid fishes to support this hypothesis (e.g., Britz and Conway 2009; Britz et al. 2009; Conway et al. 2017c), as of yet there are few examples of progenetic miniatures from other groups of fishes.

With maximum recorded sizes of 18.7 mm SL (*B.hutchinsi*) and 21.0 mm SL (*B.algicola*), the two species of *Barryichthys* are clearly miniature species (sensu Weitzman and Vari 1988) and some of the smallest gobiesocids described to date. Female individuals of *B.hutchinsi* and *B.algicola* as small as 14.2 mm SL and 17.2 mm SL, respectively, contain eggs demonstrating that they are mature and capable of reproduction at these small sizes. The high number of reductive characters exhibited by the two species of *Barryichthys*, including the absence of much of the cephalic sensory system and the lower gill-arch skeleton, are exceptional among the disc-bearing gobiesocids and may be attributed to targeted developmental truncation, at least within these character complexes. In stark contrast to these reductions, the adhesive disc of *Barryichthys* exhibits unusual, elongate papillae that are unique to this taxon amongst the disc bearing gobiesocids and may offer another, though less striking, example of the link between miniaturisation and morphological novelty from the world of fishes and the first from the Gobiesocidae.

### Comparative material

*Parvicrepisparvipinnis* – *New South Wales.*AMS I.16233-009, 2, Dee Why, Long Reef, 12 January 1972. – AMS I.166467-012, 1, 16.0 mm SL; Minnie Waters, 14 February 1965. – AMS I.16915-002, 1, 13.4 mm SL; Clovelly Pool, 30 March 1967. – AMS I.34582-001, 16, 8.0-25.4 mm SL; Nadgee, north side of Black Head, 08 June 1970. – AMS I.44125-041, 2, 19.7 mm SL; Broken Bay, North side of Lion Island, 09 May 2007. – AMS I.43799-001, 1, 18.4 mm SL; Bellambi, 14 February 2006. – AMS I.45027-038, 1, 8.5 mm SL; Mollymock, Jones Beach. – AMS I.45630-057, 8, 11.5-18.5 mm SL; Bendalong, north of boat ramp, 14 March 2011. – AMS I.45631-032, 1, 17.0 mm SL; Monument Beach, 15 March 2011. – AMS I.45935-001, 1, 12.1 mm SL; north of Tathra, south of Baronda Head, 05 April 2008. – AMS I.45633-077, 9, 10.0-15.4 mm SL; Washerwomans Beach, 16 March 2011. – AMS I.46788-001, 1, 17.0 mm SL; Ulladulla, 2012. – AMS I.46923-001, 1, 12.0 mm SL; Burrill Rocks, south of Ulladulla, 15 May 2013. – TCWC 17169.01, 40 (4 C&S, 1 CT [https://doi.org/10.17602/M2/M30713]), 14.0–23.0 mm SL; Forresters Beach, 22 February 2015. *South Australia.*AMS I.20175-008, 1, 17.2 mm SL; Kangaroo Island, Admirals Arch, 07 March 1978. *Tasmania.*AMS I.17555-003, 4 (3 male, 1 female), 12.0–19.4 mm SL; The Gardens, 6 December 1972. – AMS I.46787-002, 2, 17.0–18.0 mm SL; Coles Bay, 2012. *Victoria.*AMS I.16981-001, 16, 10.3–22.6 mm SL; Bell’s Beach AMS I.16984-004, 2, 12.7–12.8 mm SL; Anglesea, 19 March 1972. – AMS I.16988-001, 2, 20.6–22.5 mm SL; Children’s Cove, 22 March 1972.

## Supplementary Material

XML Treatment for Barryichthys

XML Treatment for Barryichthys hutchinsi

XML Treatment for Barryichthys algicola
